# Conditioned Medium from Estradiol-Primed Macrophages Mitigates Adjuvant-Induced Arthritis in Rats

**DOI:** 10.34172/apb.025.45304

**Published:** 2025-10-11

**Authors:** Farshad Yadollahi, Seyyed Meysam Abtahi Froushani, Rahim Hobenaghi

**Affiliations:** ^1^Department of Microbiology, Faculty of Veterinary Medicine, Urmia University, Urmia, Iran; ^2^Department of Pathobiology, Faculty of Veterinary Medicine, Division of Pathology, Urmia University, Urmia, Iran

**Keywords:** Rheumatoid arthritis, Estradiol, Macrophages, Conditioned medium, Immunotherapy

## Abstract

**Purpose:**

Macrophages with an anti-inflammatory phenotype are critical for resolving inflammation and preventing chronic tissue injury. Estradiol is known to promote this favorable macrophage profile. This study evaluated the therapeutic potential of the secretome, delivered as conditioned medium, from estradiol-treated macrophages in experimental rheumatoid arthritis (RA) in Wistar rats.

**Methods:**

Rheumatoid arthritis was induced in Wistar rats using complete Freund’s adjuvant. Animals were assigned to five groups: healthy controls, arthritic rats receiving vehicle, arthritic rats treated with prednisolone, arthritic rats treated with conditioned medium from untreated macrophages, and arthritic rats treated with conditioned medium from estradiol-exposed macrophages. The lyophilized media were administered intraperitoneally on days 4, 12, and 20 post-induction; the study ended on day 24.

**Results:**

Conditioned medium from estradiol-treated macrophages exhibited significantly higher levels of anti-inflammatory mediators such as interleukin-10 (IL-10), transforming growth factor-beta, and indoleamine 2,3-dioxygenase, along with increased messenger RNA expression of regulatory genes including early growth response 2 and mannose receptor. In vivo, this treatment notably reduced arthritis severity and improved weight gain compared to medium from untreated macrophages. These effects correlated with a marked decrease in antigen-specific proliferation and serum levels of inflammatory markers such as C-reactive protein (CRP), myeloperoxidase (MPO), nitric oxide (NO), IL-1, and tumor necrosis factor-alpha. Additionally, bone-destructive factors like receptor activator of nuclear factor kappa-B ligand (RANKL) and matrix metalloproteinase-9 (MMP-9) were significantly downregulated in treated rats.

**Conclusion:**

The conditioned medium derived from estradiol-treated macrophages, enriched with anti-inflammatory and regulatory components, presents a promising cell-free therapeutic strategy for immunotherapy in RA.

## Introduction

 One of the autoimmune diseases is rheumatoid arthritis (RA) characterized by joint inflammation, affecting approximately 1% of the global population. It is 2 to 3 times more common in women than men and can manifest at any age.^1,2^ The disease typically begins with progressive inflammation in multiple joints of the hands and feet, leading to pain and restricted movement.^3-6^ If the disease is not well controlled, it may cause extra-articular complications such as small vessel vasculitis, keratitis, pulmonary granulomas, as well as pericarditis and pleuritis.^7^ Some patients do not respond well to current treatments due to the disease’s complex immunopathology, and these treatments can have serious side effects. Consequently, research is ongoing to find new therapeutic approaches for these patients.^8^

 While the immune system is essential for survival and health, its potential for harm highlights the complexity and importance of maintaining a balanced immune response. This “double-edged sword” property illustrates both the advantages and risks associated with immune activity.^9^ CD4 + lymphocytes and macrophages (MQ) are crucial in immunopathological conditions like RA; they mediate and regulate immune responses, making them vital for understanding these processes.^10^ Macrophages are versatile and critical for both immediate defense against pathogens and for shaping the longer-lasting adaptive immune response. The immune system’s characteristic double-edged sword effect is primarily due to the specialized function of macrophages, which can be activated to initiate either a restorative, anti-inflammatory (M2) response or an aggressive, inflammatory (M1) reaction.^9,11-13^ Anti-inflammatory macrophages (M2) are essential for a balanced immune system, supporting tissue repair and regeneration. They can transition into M1 macrophages when facing threats like pathogens or abnormal cells. While this transition is important, an excessive M2 macrophage response can cause complications such as persistent infections, fibrosis, allergic reactions, and tumor growth.^9,14^ In contrast, M1 macrophages are mainly linked to autoimmune responses, atherosclerosis, and chronic inflammation, underscoring their varied roles in immune-related disorders.^14,15^

 Many macrophage functions are mediated by soluble factors. Macrophage-conditioned medium (MφCM) refers to the culture medium enriched with factors secreted by macrophages during in vitro growth after stimulation. When cultured, macrophages release bioactive factors such as chemokines and cytokines, as well as extracellular matrix components and growth factors into the medium.^16,17^ MφCM provides a powerful model for understanding the myriad ways in which these cells communicate and coordinate immune responses. MφCM research can help uncover potential therapeutic targets for enhancing immunity or suppressing unwanted inflammation in various diseases.^18,19^ MφCM has been utilized in the treatment and experimental modeling of various diseases, particularly in cancer research and immune response studies.^19-21^

 17β-Estradiol (E2) is a potent estrogen that influences macrophage function and polarization through estrogen receptors (ERs), primarily ERα and ERβ. It impacts immune responses, especially in asthma and autoimmune diseases. Female-derived macrophages demonstrate higher M2 polarization due to estradiol, enhancing anti-inflammatory responses compared to male-derived macrophages.^22^ Estradiol has been shown to affect the polarization of macrophages, promoting an anti-inflammatory M2 phenotype while inhibiting the pro-inflammatory M1 phenotype.^23,24^ Estradiol treatment has also been reported to increase anti-inflammatory markers like interleukin 10 (IL-10) and reduce pro-inflammatory cytokines such as IL-6 and TNF-α in macrophages. This modulation may help improve immunopathological conditions like RA.^23,25^ Despite the promising potential of conditioned medium derived from E2-primed macrophages in managing RA, much research is still needed to discover its therapeutic benefits. This research seeks to address this gap by focusing on the effects of conditioned medium obtained from E2-pulsed peritoneal macrophages on controlling experimental RA in Wistar rats.

## Material and Methods

###  Macrophage isolation and conditioned medium preparation

 Peritoneal macrophages were isolated by injecting 20 mL of cold phosphate-buffered saline (PBS) (4 °C) into peritoneal cavity of Wistar rats as previously described.^13^ To begin, peritoneal fluid collection was performed, followed by centrifugation for 10 minutes at 600 g (4 °C). The cellular pellets were then twice rinsed with PBS and reconstituted in DMEM with 10% heat-inactivated fetal bovine serum (FBS). For optimal macrophage adherence, a cellular suspension containing 2 × 10^6^ viable cells/mL was pre-incubated for 40 minutes in 48-well microplates (37 °C), maintaining a humidified environment with 5% CO2. Unadhered cells were eliminated by triple washing with cold PBS (4 °C). Cell viability, assessed through trypan blue exclusion, consistently remained above 96%.

 Afterward, macrophages were treated with either 0 or 100 nM 17β-estradiol for 24 hours. After the medium was aspirated, the cells underwent three washes with PBS. Subsequently, the macrophages were then maintained in serum-free DMEM for another 24 hours. Conditioned medium was harvested, subjected to centrifugation at 300 g for 10 minutes, and subsequently passed through a 0.2-μm filter to remove cellular debris.

 Using ELISA kits from Bender MedSystems, Austria, and according to the manufacturer’s instructions, the concentrations of TGF-β and IL-10 in the conditioned medium were determined. After isolating the conditioned medium, macrophages yielded total RNA, which was then extracted using the Trizol method and converted into complementary DNA. The mRNA expression of mannose receptor (MR) and early growth response gene-2 (EGR2) was analyzed, with GAPDH serving as an internal control. For quantification of target gene mRNA levels, SYBR green mix was used, with results reported as fold change (2^−ΔΔCt^) from at least three distinct experiments. The amplification primer sequences are provided in Table 1.

**Table 1:** The sequence of primers

**Gene**	**Forward sequence**	**Reverse primer**
MR	5'-ATGGCCTTCCTGGTGCTCT-3'	5'-TCAGGCACAGCTTCCACATC-3'
EGR2	5'-CAGCCGAGCCATGAACATC-3'	5'-GCTGGTGTTGGTGTTGATG-3'
RANKL	5'-ATGCGGTGAGCTACAGGATG-3'	5'-TTCAGGAGGATTGAGCTGGA-3'
MMP9	5'-AGCACTGTGTGCCTTTACCC-3'	5'-CCAGCCAGTCTGAGTCTTCA-3'
GAPDH	5'-GACAGTCAGCCGCATCTTCT-3'	5'-TGTAGTTGAGGTCGGTGTGA-3'

 Additionally, indoleamine-2,3-dioxygenase (IDO) activity was assessed by a kynurenine detection assay, as described previously.^9^ Briefly, 100 μL of sample received 50 μL of trichloroacetic acid (30%), after which the mixture was vortexed and centrifuged for 5 minutes at 10,000 × g. Subsequently, in a 96-well microtiter plate, 75 μL of the resulting supernatant was intermixed with 75 μL of Ehrlich’s reagent (containing 100 mg p-dimethylaminobenzaldehyde in 5 mL glacial acetic acid). Measurement of optical density was performed at 492 nm utilizing a microplate reader. To ascertain unknown concentrations, a standard curve encompassing kynurenine concentrations from 0 to 100 μM was employed.

 For the animal study phase, the isolated conditioned media were lyophilized via a Christ Alpha1-2 LD Plus freeze dryer (Germany) and were subsequently enriched 50-fold from their initial concentration.

###  Animals

 Male Wistar rats, weighing 160-180 g, were procured from the Faculty of Veterinary Medicine at our university. They were maintained under standardized conditions (55% ± 5% humidity, 20 °C-25 °C and 12 h light/dark cycle) with unrestricted access to food and water, in accordance with the Helsinki Convention and Iranian Ministry of Health regulations.

###  RA induction, monitoring, and treatment

 Induction of RA involved the intradermal injection of 0.1 mL of complete Freund’s adjuvant (CFA), formulated with 10 mg/mL of inactivated Mycobacterium, into the hind paw. An electronic water plethysmograph facilitated the measurement of non-injected hind paw volume, specifically up to the anatomical hairline of the lateral malleolus. A scoring system was employed to assess disease severity: 4 for full leg swelling and loss of flexibility; 3 for ankle swelling; 2 for erythema and paw swelling; 1 for toe erythema; and 0 for a normal paw.^26-28^

 Three independent observers conducted assessments each morning during the study, subsequently reporting the average scores. Only the non-injected paws were evaluated for arthritis, yielding a maximum score of 12. Furthermore, the weight changes of each rat were documented every other day following immunization. Therapy commenced on day 4, coinciding with the onset of RA signs in all rats, and continued until day 24 post-induction, when the animals were sacrificed. At this stage, Animals were then randomly divided into five groups of 10 rats each: RA rats received with Vehicle, RA rats treated with lyophilized macrophage conditioned medium without E2 treatment (MφCM), RA rats treated with lyophilized conditioned medium isolated from E2-pulsed macrophages (MφCM-E2), RA rats receiving prednisolone, and healthy control rats.

 The lyophilized conditioned media employed in the animal study possessed a final protein concentration of 10 mg/mL. The administration of MφCM and MφCM-E2 was performed intraperitoneally on days 4, 12, and 20, using a volume of 1 mL for each treatment. Additionally, prednisolone (2 mg/kg) was administered daily via intraperitoneal injection throughout the treatment period. In contrast, the healthy control and vehicle-treated RA rats were given an equivalent volume of PBS to maintain comparability.

###  Serum biochemical evaluation

 Cardiac blood samples, collected before sacrifice, underwent centrifugation for 10 minutes at 4,000 rpm (4 °C). Serum was subsequently isolated and stored at -70 °C until further analysis. Utilizing ELISA kits (BD, UK), and adhering to the manufacturer’s instructions, serum TNF-α and IL-1β levels were determined. The level of Nitric oxide (NO) in the serum was measured immediately after serum separation, utilizing the Griess colorimetric method, as described previously.^29^ addition, commercial kits from (BD, UK), were employed to ascertain C-reactive protein (CRP) concentrations, as per the manufacturer’s guidelines. This comprehensive approach enabled the evaluation of the inflammatory and oxidative stress status of the serum samples through the quantification of myeloperoxidase (MPO) activity, nitric oxide (NO) levels, and CRP concentrations.

 As previously detailed, the MPO activity in isolated serum samples was evaluated.^30^ For the assay, 10 μL of serum was mixed with 80 μL of hydrogen peroxide (H2O2) and 0.75 mM of TMB solution (3,3′,5,5′-tetramethylbenzidine). The TMB solution was derived from a stock containing 150 mM phosphate buffer at pH 5.4, 2.9 mM TMB and 14.5% DMSO. Following this, the mixture underwent a 5-minute incubation at 37 °C to facilitate the reaction. Reaction termination was achieved by adding 50 μL of 2 M sulfuric acid to each well of the assay plate. After an additional 5 minutes, the absorbance was measured at 450 nm using an ELISA reader. MPO activity results were quantified and expressed in units per liter (U/L).

###  Splenocyte proliferation assay

 In the splenocyte proliferation assay, spleens from each rat were dissected and placed in 5 mL of DMEM containing 10% FBS. Cellular material was isolated by passage through a 100 µm nylon mesh and subjected to centrifugation at 200 g for 10 minutes. To deplete red blood cells, ACK-lysing buffer was introduced to the pellet. In a 96-well plate, cells were prepared as a 100 µL suspension (2 × 10^5^ cells/well) and then challenged with 100 μg/mL whole mycobacterial antigen (*Mtb*). Each experiment was performed in triplicate. After 72 hours, 25 µL/well of MTT solution (5 mg/mL) was supplemented to the culture 4 hours before its conclusion. Lymphocytes were centrifuged for 10 minutes at 1000 g, and DMSO (150 µL) was added for the dissolution of formazan crystals. Absorbance at 570 nm was recorded, and the stimulation index was calculated by applying the formula: [OD (with *Mtb*) - OD (blank)] / [OD (without *Mtb*) - OD (blank)].^10^

###  Determination of the mRNA expression of MMP-9 and RANKL

 Quantitative real-time RT-PCR (qRT-PCR) necessitated the extraction of total RNA from ankle joint tissue using liquid nitrogen pulverization followed by the Trizol method, and its subsequent conversion into complementary DNA. The mRNA expression of MMP-9 (matrix metalloproteinase-9) and RANKL (receptor activator of nuclear factor kappa-B ligand) was analyzed, with GAPDH serving as an internal control. For the quantification of target gene mRNA levels, SYBR Green mix was employed, with results expressed as fold change (2^−ΔΔCt^) derived from a minimum of three independent experiments. Table 1 provides the primer sequences used for amplification.

###  Statistical Analysis

 SPSS version 21 was utilized for statistical analysis, with data presented as Mean ± SD. Analysis of nonparametric data (arthritis index) was performed using the Kruskal-Wallis test. This was subsequently followed by Mann-Whitney U evaluation, incorporating Bonferroni adjustment. Remaining parametric data were evaluated using one-way ANOVA and Tukey’s post hoc test, with a *P* value of < 0.05 considered statistically significant.

## Results

###  Initial assessment of E2-treated macrophages: gene expression and secretion profile

**Figure 1:**
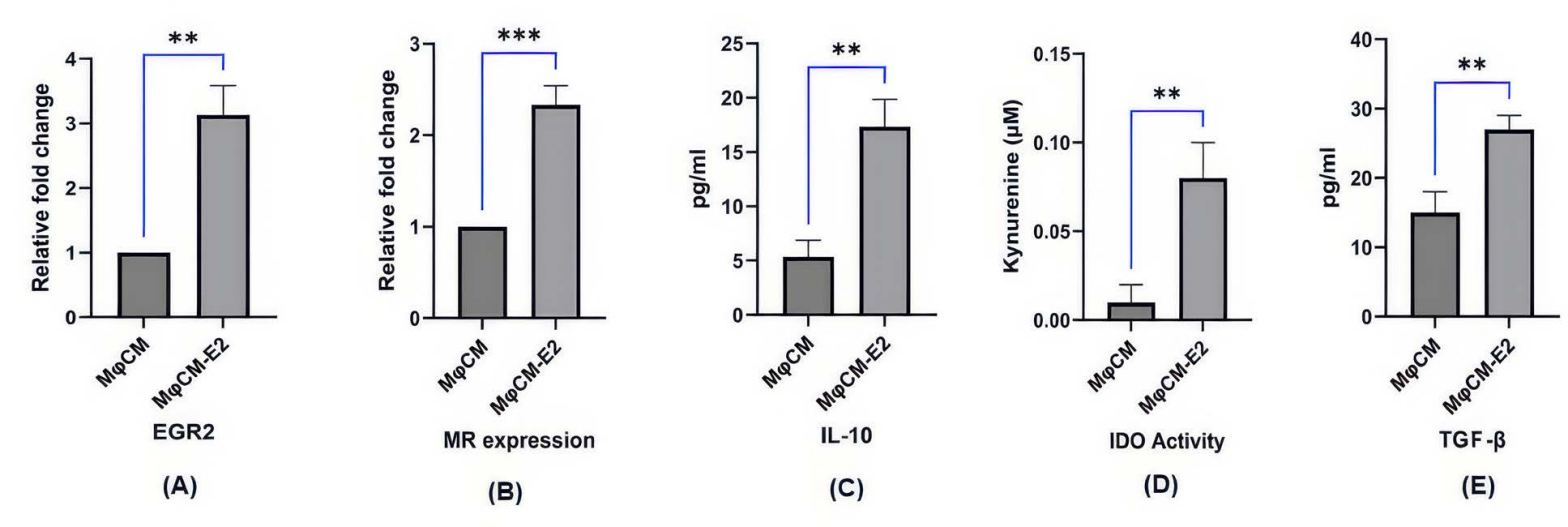
Evaluation of macrophages and their conditioned medium following 17β-estradiol treatment. Macrophages (Mφ) were treated with or without 100 nM 17β-estradiol (E2) for 24 hours to produce conditioned medium. (A, B) Real-time PCR showed increased Egr2 (Early growth response protein) and MR (Mannose Receptor) mRNA in E2-treated macrophages vs. controls. (C-E) MφCM-E2 had higher IL-10, TGF-β, and IDO (Indoleamine 2,3-dioxygenase) activity than untreated MφCM.Data are presented as mean ± SD of three independent experiments. Statistics: one-way ANOVA with Tukey's post-hoc test. (*** *P* < 0.0001, ** *P* < 0.001, ns = not significant (*P* > 0.05)). MφCM, Conditioned medium isolated from un-treated macrophages; MφCM-E2, Conditioned medium isolated from macrophages treated with 17β-estradiol (100 nM, 24 h)

 RT-PCR analysis confirmed that E2 treatment significantly augmented the mRNA expression of key genes in macrophages (Figure 1A and 1B). Specifically, EGR2 mRNA levels demonstrated a substantial 3.1-fold increase in MφCM-E2 compared to untreated MφCM (Relative fold change: 3.14 ± 0.35 vs. 1.00 ± 0.00; *P* < 0.01). Concurrently, MR mRNA expression also exhibited a significant increase of approximately 2.3-fold following E2 stimulation (Relative fold change: 2.30 ± 0.28 vs. 1.00 ± 0.00; *P* < 0.001). These findings indicate a clear transcriptional modulation by E2 towards a specific macrophage phenotype.

 Further investigation of the conditioned medium from these macrophages revealed a marked shift towards an anti-inflammatory and immunomodulatory profile. We observed a significant increase in the levels of IL-10 (17.52 ± 2.45 pg/mL) in MφCM-E2 compared to MφCM (5.26 ± 0.76 pg/mL; P < 0.01) (Figure 1C). Similarly, TGF-β levels were significantly higher in MφCM-E2 (27.29 ± 3.48 pg/mL) compared to MφCM (13.75 ± 3.42 pg/mL; P < 0.01) (Figure 1E). Furthermore, IDO activity, as quantified by the level of kynurenine, was substantially enhanced in MφCM-E2 (0.076 ± 0.01 pg/mL) relative to MφCM (0.011 ± 0.005 pg/mL), representing approximately a 7-fold increase (*P* < 0.01) (Figure 1D). These findings collectively indicate that E2 effectively polarizes macrophages towards an immunosuppressive profile, characterized by enhanced expression of regulatory genes and secretion of anti-inflammatory mediators.

###  Clinical efficacy of macrophage-conditioned medium in a rat model of rheumatoid arthritis

**Figure 2:**
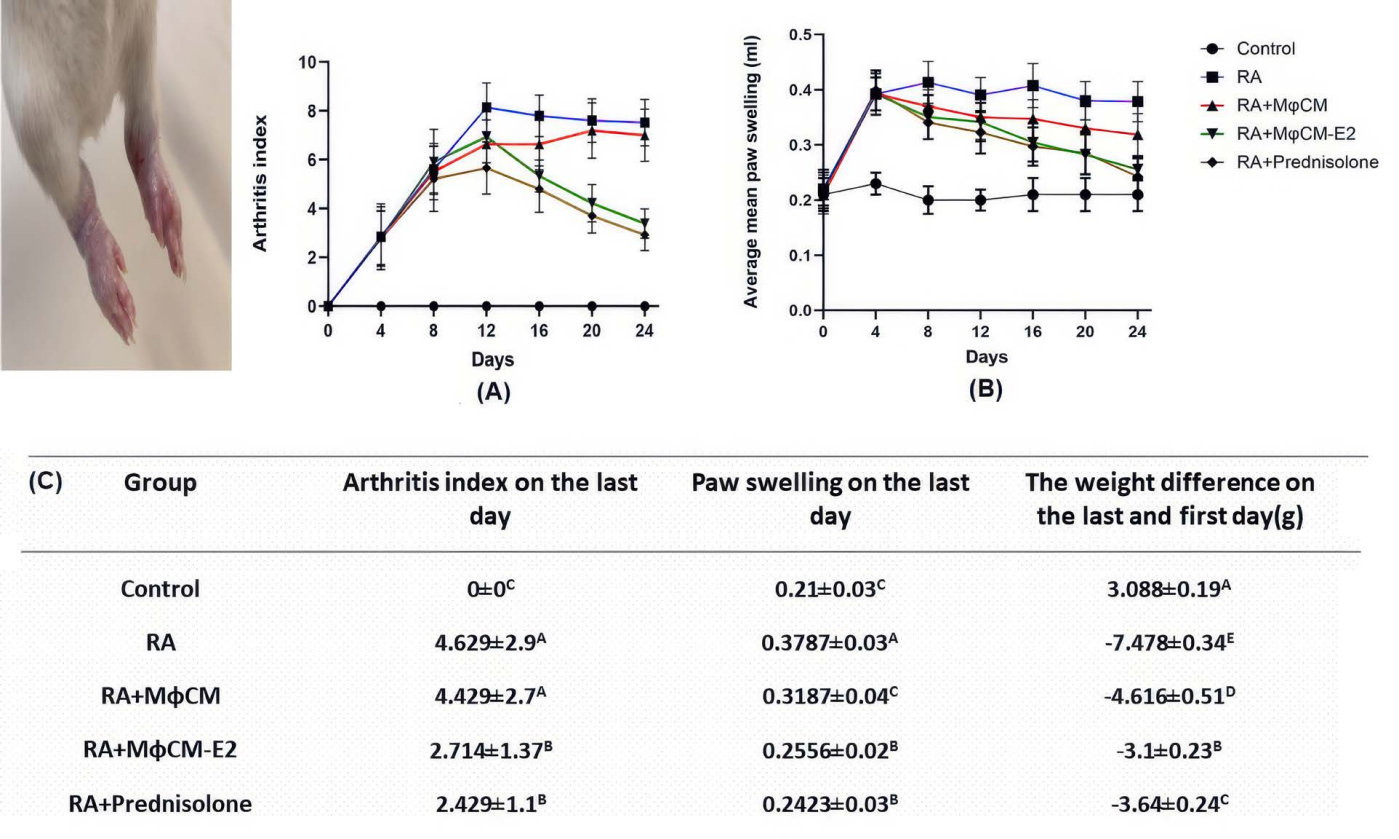
Clinical features of rheumatoid arthritis in rats following different treatments. RA was induced in rats using complete Freund’s adjuvant, and treatment started 4 days post-induction until day 24. (A, B) Arthritis index and paw swelling over time. (C) Summary table. MφCM-E2 significantly reduced the arthritis index (~41%) and paw swelling (~32%) versus untreated RA rats, similar to prednisolone. MφCM-E2 significantly improved weight gain more than untreated RA rats and prednisolone. MφCM alone improved weight gain but not clinical scores. Data are mean ± SD. Statistics: one-way ANOVA with Tukey’s post-hoc test. Letters (A, B, C, D, E) on the graphs indicate significant differences between the groups (*P* < 0.05). MφCM, Untreated macrophage conditioned medium; MφCM-E2, 17β-estradiol (100 nM, 24 h) treated macrophage conditioned medium

 In the established Complete Freund’s Adjuvant-induced arthritis model in rats, significant clinical indicators of inflammation were observed following induction Figure 2A. Treatment commenced on day 4 post-induction when the arthritis index reached ≥ 1. Untreated RA rats exhibited a progressive increase in both arthritis index and paw swelling from day 4 onwards, peaking around day 12-16 (Figure 2B) On the last day of evaluation (Day 24), the untreated RA group displayed a mean arthritis index of 4.629 ± 2.9 and an average mean paw swelling of 0.3787 ± 0.03 mm.

 Treatment with MφCM-E2 and prednisolone demonstrated effective and statistically similar reductions in several key clinical indicators of RA in rats (Figure 2C). On the last day, the MφCM-E2 group showed a mean arthritis index of 2.714 ± 1.37, representing a 41.38% reduction compared to untreated RA rats. The prednisolone group achieved a comparable arthritis index of 2.429 ± 1.1, showing a 47.47% reduction (*P*< 0.05 for both MφCM-E2 and prednisolone vs. RA group; non-significant) ns (between MφCM-E2 and prednisolone). Similarly, hind paw swelling on the last day was significantly reduced in the MφCM-E2 group to 0.2556 ± 0.02 mm (a 32.51% reduction from RA group) and in the prednisolone group to 0.2423 ± 0.03 mm (a 36.02% reduction from RA group) (*P* < 0.05 for both MφCM-E2 and prednisolone vs. RA group; ns between MφCM-E2 and prednisolone). Conversely, treatment with MφCM alone showed no significant impact on alleviating the severity of clinical symptoms, with an arthritis index of 4.429 ± 2.7 and paw swelling of 0.3187 ± 0.04 mm on the last day, remaining statistically comparable to vehicle-treated RA rats (ns vs. RA group for both parameters).

 Furthermore, RA induction consistently led to notable weight loss in affected animals, with the untreated RA group experiencing an average weight difference of -7.478 ± 0.34 g between the last and first day (Figure 2C). Encouragingly, all treated animal groups exhibited improved weight gain trajectories compared to untreated RA rats. The MφCM-E2 group showed particularly robust improvement in weight gain, with a mean weight difference of -3.1 ± 0.23 g, which outperformed the prednisolone group (mean weight difference of -3.64 ± 0.24 g; *P* < 0.05 vs. prednisolone). Notably, while treatment with MφCM did not demonstrate significant improvement in clinical symptoms as previously noted, it was still effective in improving weight gain in affected rats, with a mean weight difference of -4.616 ± 0.51 g (P < 0.05 vs. untreated RA rats). This suggests a distinct systemic effect of MφCM beyond direct anti-inflammatory action on joints.

###  Biochemical markers of inflammation and oxidative stress

**Figure 3:**
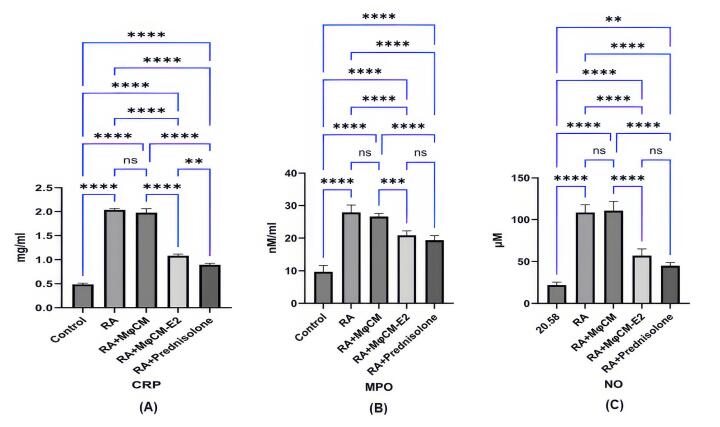
Assessment of biochemical factors in the sera of RA rats following treatment. (A) to (C) Bar graphs displaying the serum concentrations of C-reactive protein (CRP), myeloperoxidase (MPO), and nitric oxide (NO). Untreated RA rats showed significantly elevated levels of these markers compared to healthy controls. Both MφCM-E2 and prednisolone treatments significantly lowered these levels in RA rats. Prednisolone significantly reduced CRP compared to other treatment groups. MφCM alone had no significant effect on these factors. Values are mean ± SD. Statistics: one-way ANOVA with Tukey’s post-hoc test. (**** *P* < 0.00001, ** *P* < 0.001, ns: non-significant (*P* > 0.05)). MφCM, Untreated macrophage conditioned medium; MφCM-E2, 17β-estradiol (100 nM, 24 h) treated macrophage conditioned medium

 Biochemical analysis of serum samples from the CFA-induced arthritis model revealed a significant elevation in systemic inflammatory and oxidative stress markers in untreated RA rats compared to healthy controls (Figure 3A, 3B and 3C). CRP levels were markedly increased in RA rats (2.03 ± 0.04 mg/mL) compared to control (0.50 ± 0.03 mg/mL; *P* < 0.00001). Similarly, MPO levels rose significantly in RA rats (27.87 ± 2.01 nM/mL vs. 9.87 ± 0.77 nM/mL in control; *P* < 0.00001), and NO levels were substantially augmented (107.03 ± 6.75 µM vs. 20.58 ± 2.59 µM in control; *P* < 0.00001).

 Treatment with both MφCM-E2 and prednisolone effectively mitigated these elevations in RA rats. Specifically, MφCM-E2 treatment resulted in a significant reduction of CRP levels to 1.09 ± 0.05 mg/mL (*P* < 0.00001 vs. RA), while prednisolone reduced CRP to 0.90 ± 0.05 mg/mL (*P* < 0.00001 vs. RA). Prednisolone demonstrated a significantly stronger effect in reducing CRP levels compared to MφCM-E2 (*P* < 0.001). Notably, MφCM-E2 treatment achieved a greater reduction in MPO levels, bringing them down to 20.27 ± 0.78 nM/mL (*P* < 0.00001 vs. RA group, and ns vs. prednisolone group (19.49 ± 0.76 nM/mL)). In contrast, prednisolone led to a more pronounced reduction in NO levels (45.33 ± 3.49 µM; *P* < 0.00001 vs. RA) compared to MφCM-E2 (55.53 ± 2.76 µM; *P* < 0.00001 vs. RA, but ns vs. prednisolone) (Figure 3A, 3B and 3C). The use of MφCM alone did not yield a statistically significant effect on reducing serum levels of CRP (1.98 ± 0.03 mg/mL), MPO (26.69 ± 1.13 nM/mL), or NO (108.97 ± 3.20 µM) compared to untreated RA rats (ns for all parameters).

###  Impact on pro-inflammatory cytokines

**Figure 4:**
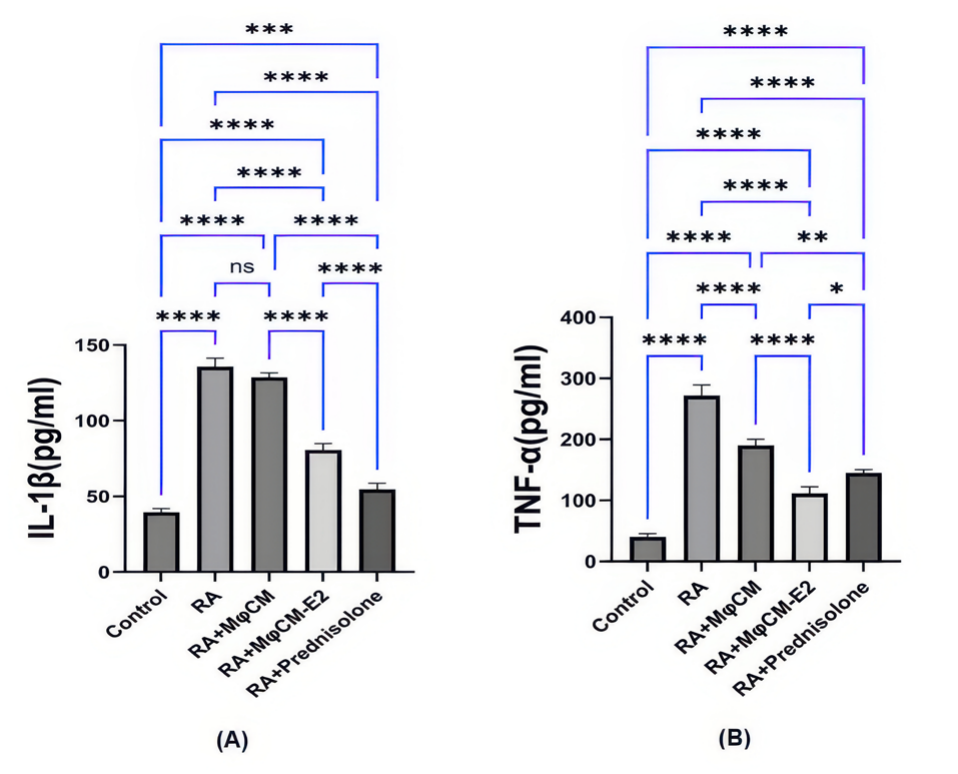
Serum levels of inflammatory Cytokines in RA Rats After Treatment. (A) and (B) Bar graphs showing the serum concentrations of IL-1β and TNF-α, respectively. In RA rats, MφCM-E2 and prednisolone significantly lowered serum IL-1β and TNF-α. MφCM-E2 reduced TNF-α more than prednisolone, while prednisolone reduced IL-1β more than MφCM-E2. MφCM lowered TNF-α, but to a lesser extent. Values are expressed as as mean ± S.D. Statistics: one-way ANOVA with Tukey’s post-hoc test. (**** *P* < 0.00001, *** *P* < 0.0001, ** P < 0.001, * *P* < 0.05,ns:non-significant (*P* > 0.05)). MφCM, Untreated macrophage conditioned medium; MφCM-E2, 17β-estradiol (100 nM, 24 h) treated macrophage conditioned medium

 As illustrated in Figure 4, treatment with MφCM-E2 and prednisolone led to significant decreases in the serum levels of key pro-inflammatory cytokines, IL-1β and TNF-α, in CFA-challenged rats. Serum IL-1β levels, which were significantly elevated in untreated RA rats (135.22 ± 5.86 pg/mL vs. 40.89 ± 2.96 pg/mL in healthy controls, *P* < 0.00001), were significantly reduced by both MφCM-E2 (82.12 ± 7.97 pg/mL; *P* < 0.00001 vs. RA) and prednisolone (53.38 ± 4.54 pg/mL; *P* < 0.00001 vs. RA). Data analysis revealed that prednisolone resulted in a more pronounced reduction in serum IL-1β levels than MφCM-E2 (*P* < 0.00001). Treatment with MφCM alone (129.83 ± 6.07 pg/mL) showed no significant effect on IL-1β levels compared to untreated RA (ns) (Figure 4A).

 Similarly, serum TNF-α levels, significantly elevated in untreated RA rats (273.84 ± 19.34 pg/mL vs. 39.56 ± 2.93 pg/mL in healthy controls, *P* < 0.00001), exhibited the most substantial reduction in the RA group treated with MφCM-E2, decreasing to 112.98 ± 12.08 pg/mL (*P* < 0.00001 vs. RA). This reduction by MφCM-E2 was significantly greater than that achieved by prednisolone (reduced to 143.76 ± 12.63 pg/mL; *P* < 0.001 vs. MφCM-E2). Treatment with MφCM alone also lowered TNF-α levels to 192.56 ± 21.08 pg/mL (*P* < 0.00001 vs. RA), although the extent of this reduction was statistically less significant compared to both MφCM-E2 (*P* < 0.00001 vs. MφCM) and prednisolone (*P*< 0.001 vs. MφCM) groups (Figure 4B).

###  Modulation of antigen-specific splenocyte proliferation

**Figure 5:**
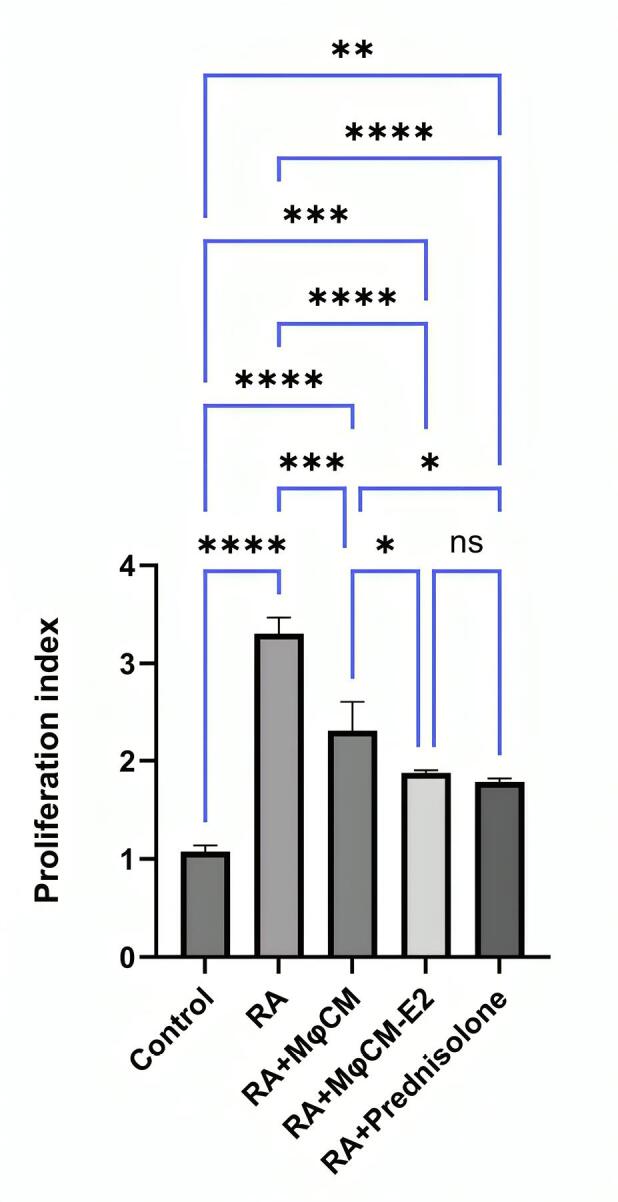
Evaluation of antigen-specific splenocyte proliferation in treated RA rats. The proliferation index of antigen-specific splenocytes was assessed in response to treatment. Untreated RA rats exhibited a significantly higher proliferation index compared to healthy controls. Both MφCM-E2 and prednisolone similarly reduced lymphocyte proliferation. RA rats treated with MφCM showed a less effective reduction compared to MφCM-E2 and prednisolone. Results are expressed as mean ± SD. Statistics: one-way ANOVA with Tukey’s post-hoc test. (**** *P* < 0.00001, *** *P* < 0.0001, ** *P* < 0.001, * *P*< 0.05). MφCM, Untreated macrophage conditioned medium; MφCM-E2, 17β-estradiol (100 nM, 24 h) treated macrophage conditioned medium

 The intensity of antigen-specific splenocyte proliferation, a measure of adaptive immune response, was significantly increased in CFA-challenged rats compared to healthy controls (Figure 5). The untreated RA group exhibited a mean proliferation index of 3.22 ± 0.17, which was significantly higher than the control group (1.08 ± 0.05; *P* < 0.00001).

 Encouragingly, all therapeutic interventions resulted in a notable reduction in the splenocyte proliferation index compared to untreated RA rats. Specifically, groups treated with MφCM-E2 (proliferation index of 1.89 ± 0.07) and prednisolone (proliferation index of 1.83 ± 0.04) were statistically equally effective in decreasing the lymphocyte proliferation index (*P* < 0.00001 vs. RA for both, and ns between them). RA rats treated with MφCM showed a significant reduction in proliferation index to 2.29 ± 0.28 (*P*< 0.0001 vs. RA), but this effect was statistically less effective than both MφCM-E2 (*P* < 0.05 vs. MφCM) and prednisolone groups (*P *< 0.05 vs. MφCM) (Figure 5).

###  Impact on cartilage and bone degradation markers in ankle joint tissue

 As depicted in Figure, the mRNA expression of key enzymes involved in cartilage and bone degradation, RANKL and MMP-9, exhibited significant reductions in the ankle joint tissue of rats with arthritis that received MφCM-E2 and prednisolone, compared to untreated RA rats.

**Figure 6:**
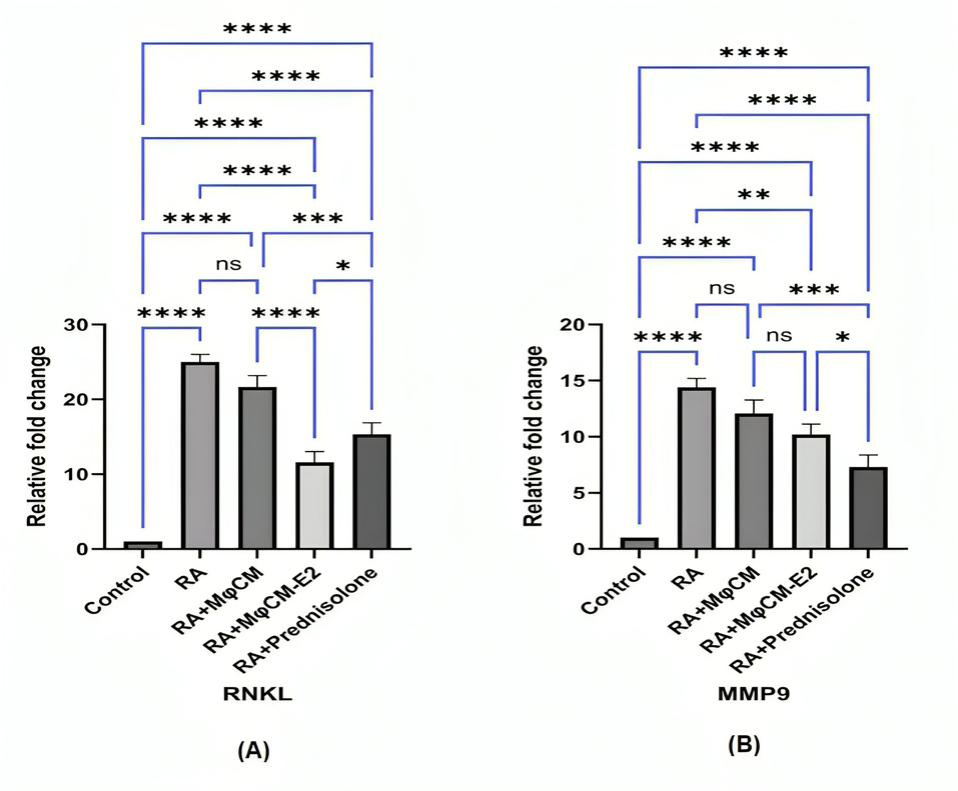
Determination of mRNA expression of RANKL and MMP-9 in ankle joint tissue of RA rats. (A) and (B) Bar graphs showing the mRNA expression of RANKL (Receptor Activator of Nuclear Factor Kappa-B Ligand) and MMP-9 (Matrix Metalloproteinase-9), respectively. In arthritic rats, MφCM-E2 and prednisolone significantly reduced RANKL and MMP-9 mRNA expression compared to untreated rats. MφCM-E2 reduced RANKL mRNA more than prednisolone, but prednisolone reduced MMP-9 mRNA more than MφCM-E2. MφCM alone did not significantly reduce these mRNAs. Findings are expressed as mean ± S.D. Statistics: one-way ANOVA with Tukey’s post-hoc test. (**** *P* < 0.00001, *** *P* < 0.0001, ** *P* < 0.001, * *P* < 0.05, ns: non-significant (*P* > 0.05)). MφCM, Untreated macrophage conditioned medium; MφCM-E2, 17β-estradiol (100 nM, 24 h) treated macrophage conditioned medium

 In the RA group, RANKL mRNA relative fold change was 25.04 ± 1.05, significantly higher than control (1.00 ± 0.00; *P* < 0.00001). Statistically, the effect of MφCM-E2 treatment was more pronounced in reducing mRNA expression of RANKL in the ankle joint tissue (Relative fold change: 11.08 ± 0.69; *P*< 0.00001 vs. RA) compared to prednisolone treatment (Relative fold change: 15.24 ± 1.03; *P*< 0.00001 vs. RA, but *P*< 0.05 vs. MφCM-E2) (Figure 6A).

 Conversely, the opposite effect was noted for mRNA expression of MMP-9. In the RA group, MMP-9 mRNA relative fold change was 14.59 ± 0.81, significantly higher than control (1.00 ± 0.00; *P* < 0.00001). Here, prednisolone induced a greater reduction (Relative fold change: 6.93 ± 0.69; *P* < 0.00001 vs. RA) than MφCM-E2 (Relative fold change: 10.25 ± 0.70; *P* < 0.001 vs. RA, but *P* < 0.05 vs. prednisolone) (Figure 6B). Ultimately, treatment with MφCM alone in rats with arthritis did not yield statistically significant reductions in any of these mRNAs in the ankle joint tissue (RANKL relative fold change: 21.65 ± 1.02; MMP-9 relative fold change: 12.09 ± 0.79; ns for both vs. untreated RA rats) (Figure 6A and 6B).

## Discussion

 Immunotherapy, particularly using cell therapy, offers a more targeted approach compared to traditional immunosuppressive therapies, potentially leading to fewer side effects and improved efficacy. It aims to modulate rather than completely suppress the immune system, allowing for better management of autoimmune diseases.^31,32^ However, cell therapy often necessitates personalized treatment for each patient. This approach involves the use of a clean room and the re-transplantation of cells into the host, making it a multi-step and costly process. The production of a conditioned medium rich in anti-inflammatory mediators from macrophages following estrogen pulsing and lyophilization, under conditions akin to those of the present study, could offer a commercializable, low-risk, low-cost, and effective solution for alleviating the symptoms of inflammatory autoimmune diseases, including RA. Lyophilization of conditioned medium for immunotherapy serves to improve stability, shelf-life, and convenience, all while preserving the biological activity of important components, ultimately supporting more effective and practical use in clinical and research contexts.^33^

 Previous literature indicates that estradiol promotes M2/anti-inflammatory macrophage formation through a combination of receptor-mediated signaling, modulation of cytokine production, transcription factor activation (like STAT3 and p38 MAPK), changes in metabolic processes, and potentially through the regulation of microRNAs (like downregulating miR-155) and intercellular communication.^34,35^ Estrogen boosts macrophage to produce of IDO, an enzyme that metabolizes tryptophan, a key component in immune regulation. Increased IDO activity reduces inflammation and fosters immune tolerance by depleting tryptophan necessary for T-cell growth. IDO production is a hallmark of M2/anti-inflammatory macrophages.^36^ Additionally, studies indicate that estradiol increases the production of other M2 markers, such as TGF-β, IL-10, and arginase.^22^ NF-κB and STAT3 modulate macrophage M2 polarization.^37-39^ The E2/ERα complex activates STAT3 and suppresses NF-κB, boosting IL-10 production and M2 polarization in macrophages.^40,41^ In this context, our current study revealed a notable increase in the levels of TGF-β, IL-10, and IDO activity in the conditioned medium obtained from E2-treated Mφ compared to that from untreated Mφ.

 Egr2 and MR (CD206) are crucial for the induction and maintenance of anti-inflammatory M2 macrophage polarization. High levels of Egr2 and MR enable macrophages to maintain their plasticity, allowing them to adapt and respond effectively to changes in the inflammatory environment. Conversely, low levels of Egr2 and MR are linked to non-responsiveness, which can impair the immune response.^42,43^ Our results showed that E2 treatment increased the mRNA expression of Egr2 and MR in macrophages compared to untreated ones. Overall, the results of the analysis of the conditioned medium and the RT-PCR findings suggest that treating macrophages with E2 effectively induced an anti-inflammatory phenotype similar to the M2 macrophage phenotype.

 The animal studies phase of the current research indicated that while the use of MφCM did not significantly alleviate the severity of clinical symptoms in RA rats, treatment with MφCM-E2 and prednisolone effectively and comparably reduced clinical indices, such as the arthritis index and hind paw swelling in RA rats, thereby diminishing their suffering. The beneficial results of cell therapy using E2-treated mesenchymal stem cells (MSCs) have been noted in two recent scientific sources. A previous study revealed that exposing MSCs to E2 at a concentration of 100 nM for 24 hours significantly enhanced the suppression of T lymphocyte proliferation, increased IDO, IL-10, NO, and TGF-β production, and upregulated CXCR4 and CCR2 mRNA expression in MSCs. This treatment showed greater efficacy in alleviating RA severity in rats compared to MSCs alone.^30^ In another experiment, it was observed that treatment with E2-pulsed MSCs led to a reduction in the cumulative clinical score and a significant enhancement in neuropathology in rats with an experimental form of multiple sclerosis, compared to treatment with un-pulsed MSCs.^44^

 MPO, an enzyme produced by neutrophils, plays a crucial role in oxidative stress and inflammation, serving as a biomarker for various conditions, including RA. Elevated serum MPO levels in RA patients correlate with increased disease activity and other inflammatory markers, indicating its potential for evaluating disease progression and treatment efficacy.^45,46^ Similarly, the serum level of NO is significantly higher in RA patients and correlates with disease severity.^47,48^ The data obtained from the current survey indicated that, in contrast to treatment with MφCM, which did not affect MPO and NO levels, MφCM-E2 demonstrated greater effectiveness than prednisolone in reducing both MPO and NO levels. Additionally, CRP, an inflammatory marker produced in response to cytokines, is also elevated in RA and correlates with disease activity;^49,50^ however, MφCM-E2 treatment effectively lowers CRP levels, unlike MφCM. Collectively, these findings highlight the significance of MPO, NO, and CRP as biomarkers in monitoring RA and the potential of MφCM-E2 treatments in managing inflammation. Recent experiments indicate that E2-pulsed MSCs have a similar efficacy to prednisolone in reducing RA symptoms and lowering levels of CRP, RF, and NO.^30^ Additionally, a notable decrease in serum levels of NO and MPO was found in rats with experimental autoimmune encephalomyelitis (EAE) treated with E2-pulsed MSCs compared to those receiving untreated MSCs.^44^

 IL-1 and TNF-α significantly promote inflammation in joint synovial tissue, recruiting more immune cells and perpetuating joint damage.^51^ Previous studies showed that MSC-conditioned media have anti-inflammatory effects by inhibiting the NF-κB and MAPK pathways, resulting in reduced activation of inflammatory mediators like IL-1 and TNF-α, thus promoting a balanced immune response.^52^ Here, we reported that treatment with MφCM-E2 significantly decreased serum IL-1β and TNF-α levels in CFA-challenged rats, whereas treatment with MφCM was effective only in reducing TNF-α levels.

 RANKL is a pivotal mediator in the pathophysiology of RA-related bone erosion through its role in promoting osteoclast differentiation and activity. RANKL expression is influenced by various inflammatory cytokines such as IL-1, IL-6 and TNF-α, which are upregulated in RA.^53^ MMP-9 exhibits a dual role in RA progression, promoting both cartilage degradation and enhanced inflammatory responses within rat ankle joints. MMP-9 expression correlates with disease severity in RA.^54^ In previous studies, mainly based on conditioned medium obtained from MSCs, it has been shown that MSC-conditioned medium significantly impacts RANKL and MMP-9 levels, thereby influencing osteoclast differentiation and activity.^55,56^ According to our results, unlike MφCM, treatment with MφCM-E2 significantly decreased RANKL and MMP-9 mRNA expression in ankle joint tissue compared to untreated rats.

## Conclusion

 In comparison to conditioned medium isolated from Mφ, pretreatment of Mφ with estradiol promotes a potent anti-inflammatory milieu in the secretome of Mφ. An in vivo study indicated that MφCM-E2 caused more profound beneficial effects in the treatment of RA in Wistar rats compared to RA rats treated with MφCM. The outcomes of treatment with MφCM-E2 were comparable to those of prednisolone. Given the excellent potential of MφCM-E2, this approach may represent a promising and beneficial strategy for RA immunotherapy. However, this survey is a preliminary study in an animal model, and further studies are required to demonstrate the efficacy of MφCM-E2 in humans with RA.

## Competing Interests

 The authors declare that they have no known competing financial interests.

## Ethical Approval

 Ethical considerations concerning the use of laboratory animals were conducted in accordance with the regulations set forth by the Iranian Ministry of Health and the Helsinki Convention, following approval from the Ethics Committee of the Faculty of Veterinary Medicine, Urmia University, Urmia, Iran.
